# Midbrain catecholaminergic neurons co-express α-synuclein and tau in progressive supranuclear palsy

**DOI:** 10.3389/fnana.2015.00025

**Published:** 2015-03-11

**Authors:** María Elena Erro Aguirre, María Victoria Zelaya, Javier Sánchez Ruiz de Gordoa, María Teresa Tuñón, José Luis Lanciego

**Affiliations:** ^1^Department of Neurology, Complejo Hospitalario de NavarraPamplona, Spain; ^2^Instituto de Investigación Sanitaria de Navarra (IDISNA)Pamplona, Spain; ^3^Brain Bank, Navarra BiomedPamplona, Spain; ^4^Department of Neuropathology, Complejo Hospitalario de NavarraPamplona, Spain; ^5^Department of Neurosciences, Center for Applied Medical Research (CIMA)Pamplona, Spain; ^6^Centro de Investigación Biomédica en Red sobre Enfermedades Neurodegenerativas (CIBERNED)Pamplona, Spain

**Keywords:** neurodegenerative diseases, atypical parkinsonian disorders, lewy bodies, multiple immunolabeling techniques, substantia nigra

## Abstract

**Objective**: To analyze the frequency and distribution of α-synuclein deposits in progressive supranuclear palsy (PSP).

**Methods**: The brains of 25 cases of pathologically confirmed PSP were evaluated with immunohistochemistry for α-synuclein and tau. Multiple immunofluorescent stains were applied to analyze the expression of tau and α-synuclein aggregates in catecholaminergic neurons. Patients’ clinical symptoms were retrospectively recorded.

**Results**: Deposits α-synuclein in the form of typical Lewy bodies (LBs) were only found in two PSP cases (8%) that fulfilled the clinical subtype of PSP known as Richardson’s syndrome (RS). LBs were present in the locus ceruleus (LC), substantia nigra pars compacta (SNc), basal forebrain, amygdala and cingulated cortex in a distribution mimicking that of Parkinson’s disease (PD). Triple-immunolabeling revealed co-expression of α-synuclein and tau proteins in some tyrosine hydroxilase (TH)-positive neurons of the LC and SNc.

**Conclusions**: There is no apparent clinical correlation between the presence of LBs in PSP. Tau protein co-aggregate with α-synuclein in catecholaminergic neurons of PSP brains suggesting a synergistic interaction between the two proteins. This is in keeping with the current view of neurodegenerative disorders as “misfolded protein diseases”.

## Introduction

Aggregation of α-synuclein in the form of Lewy bodies (LBs) is the histopathological hallmark of idiopathic Parkinson’s disease (PD) and dementia with LBs (DLB; Spillantini et al., 1997). LBs are present in the 50% of Alzheimer’s disease brains (AD; Hamilton, 2000) but they are less frequently found in other neurodegenerative diseases such as progressive supranuclear palsy (PSP; Abhinav et al., 2011). At present it is well known that protein aggregation phenomena represent a crucial pathobiological mechanism shared by neurodegenerative diseases such AD, PD, PSP and Huntington disease. Accordingly, these diseases have often been categorized as “misfolded protein diseases”, since cross-seeding of misfolded proteins apparently underlies a number of pathological events associated with different diseases (reviewed in Soto and Estrada, 2008; Cuanalo-Contreras et al., 2013; Morales et al., 2013).

PSP is the most common atypical parkinsonian disorder and is clinically characterized by prominent postural instability, vertical gaze supranuclear palsy, pseudobulbar palsy, cognitive impairment and levodopa unresponsiveness (Steele et al., 1964). The pathology of PSP is characterized by the accumulation of abnormal tau protein within neurons (neurofibrillary tangles) and glial cells (tufted astrocytes and coiled bodies) in the brain. The distribution of tau inclusions is mainly subcortical and the globus pallidus, subthalamic nucleus and substantia nigra are severely affected in most cases. Other subcortical nuclei are affected to varying degrees and cases of severe cortical degeneration have been also described (Litvan et al., 1996). Whether the presence of LBs in PSP represents a normal aging process or the coexistence with PD remains unclear (Uchikado et al., 2006).

The aim of the present study was to analyze the density and distribution of LBs in the brains of patients with PSP, to investigate the clinicopathological correlation of this association and to characterize the interaction of α-synuclein and tau in cases of PSP with LBs by means of multiple immunolabeling techniques.

## Materials and Methods

### Subjects

Twenty five cases (2005–2013) of pathologically confirmed PSP according to the revised NINDS criteria (Litvan et al., 1996) from the Navarra Biomed Brain Bank were studied. The establishment and operational rules of Navarra Biomed Bank are both under the supervision of a Local Clinical Ethical Committee in keeping with current Spanish legislation (Royal Decree 1716/2011). Informed written consent form was obtained in all cases. The clinical features of those cases with LBs were evaluated by retrospective review of medical records. The distribution of LBs was assessed with the Braak’s PD staging scheme (Braak et al., 2003). For control purposes, the mesencephalon of two age-matched individuals (both males) without any known type of neurological disease were used.

### Neuropathological Evaluation

The brains were dissected through the corpus callosum in the sagittal plane. The left cerebral hemisphere was immersed in a 10% saline solution of 10% formaldehyde for 4 weeks. According to the recommendation guide proposed by BrainNet Europe (Bell et al., 2008), multiple regions of the neocortex were examined including frontal cortex (middle frontal gyrus), superior temporal, inferior parietal, occipital and cingulate cortex, entorhinal cortex, hippocampus, amygdala, basal nucleus of Meynert, striatum, subthalamic nucleus, brainstem and cerebellum.

Routine workflow included immunohistochemical staining of 3–5 μm-thick paraffin-embedded sections, followed by counterstaining with hematoxylin-eosin.

### Immunohistochemistry

Formalin-fixed sections (3–5 mm-thick) were mounted on slides and deparaffinized. After conducting a routine antigen retrieval protocol, sections were incubated overnight with either a mouse monoclonal antibody against α-synuclein (NCL-L-ASYN; Leica Biosystems) or with a mouse monoclonal antibody anti-human PHF-TAU (clone AT-8; Thermo Scientific). The reaction product was visualized using an automated slide immunostainer (Leica Bond Max) with Bond Polymer Refine Detection (Leica Biosystems Newcastle Ltd).

For semiquantitative analysis LBs density and Lewy neurites (LN) were scored as follows: 0 = absent, + = mild, ++ = moderate, +++ = severe and ++++ = very severe (McKeith et al., 2005). In the assessment LBs brainstem type and cortical were included. A PD neuropathological stage was assigned according to the staging scheme proposed by Braak. For this measure only the distribution of LBs in any brain region was taken into account instead of the LBs density.

### Dual Colorimetric Immunohistochemistry

Briefly, a sequential dual immunostaining protocol was used for the simultaneous visualization of α-synuclein and tau. The colorimetric detection of α-synuclein was carried out firstly using a Bond Polymer Refine DAB detection kit (Leica) resulting in a brown precipitate. Next, the Bond Polymer Refine Red detection kit (Leica) was used to visualize the expression of tau protein by means of a red-colored precipitate.

### Multiple Immunofluorescence and Confocal Microscopy

Multiple immunofluorescent stains were carried out in coronal sections through the mesencephalon comprising both the substantia nigra pars compacta (SNc) and the locus ceruleus (LC). Briefly, 5 mm-thick brain blocks were postfixed for 24 h in a buffered solution containing 4% paraformaldehyde. Once fixed, sections were cryoprotected with a solution made of 20% gelatin and 2% dimethylsulphoxide (DMSO) in 0.1 M PBS, pH 7.4. Next, frozen coronal sections (40 μm-thick) were obtained in a sliding microtome and collected in the cryoprotective solution as 5 series of adjacent sections.

Free-floating sections were incubated in a cocktail of primary antisera comprising 1:200 mouse anti-synuclein (Invitrogen, Ref. 08-1215), 1:200 rabbit anti-Tau (Dako-Sigma, Ref. A0024) and 1:50 goat anti-tyrosine hydroxilase (TH) (Santa Cruz, Ref. Sc-7847) overnight at room temperature. Sections were then incubated for 90 min at room temperature in a cocktail of alexa-tagged secondary antibodies, comprising 1:200 Alexa488-coupled donkey anti-mouse IgG (Invitrogen, A21202), Alexa546-coupled donkey anti-rabbit IgG (invitrogen, Ref. A31572) and Alexa 633-coupled donkey anti-goat IgG (Invitrogen, Ref. A21082). Stained sections were finally mounted in gelatine-coated slides, air dried at room temperature in the dark, dehydrated in toluene and mounted with Entellan (Merck).

Sections were inspected in a Zeiss 510 META confocal laser-scanning microscope. To ensure appropriate visualization of the labeled structures and to avoid false positive results, the emission from the argon laser at 488 nm was filtered through a band pass filter of 505–530 nm and color-coded in green. The emission following excitation with the helium laser at 543 nm was filtered through a band-pass filter of 560–615 nm and color coded in light blue. Finally, a long-pass filter of 650 nm was used to visualize the emission from the helium laser at 633 nm and color coded in red.

## Results

### Clinical Features of the PSP Patients

The first patient was an 84-year-old woman who underwent consultation at the Neurology Department complaining of progressive gait unsteadiness, with postural imbalance and frequent falls reported over the previous year. She did not refer cognitive decline. Neurologic examination revealed facial inexpressiveness and mild hypophonia. Glabelar and sucking reflexes were present. Ocular motility was normal, generalized bradykinesia and rigidity were present with a rigid neck extension posture, deep tendon reflexes were brisk, gait was slow and unsteady with severe impairment of postural righting reflexes. Computed cranial tomography showed moderate cortical atrophy. A diagnosis of atypical parkinsonian disorder with early gait impairment suggestive of possible PSP was established and treatment with carbidopa/levodopa was initiated. One year later she referred impossibility in rising from a seated position and difficulty in language articulation. Her gait disorder progressed and she was wheelchair bound. Response to carbidopa/levodopa was absent. Moreover, a mild reduction of up gaze was found during the clinical examination. On subsequent visits, oculomotor disorder was established with blepharospasm, apraxia of eyelid closing and vertical gaze palsy. She died from aspiration pneumonia related to dysphagia 4 years and 2 months after the first visit.

The second patient was a 73 year old man complaining from memory loss and reduced talkativeness. Neuropsychological evaluation showed mild short-term memory loss, disturbance of executive functions, word finding difficulties and perseverative errors. The diagnosis of a lobar frontotemporal dementia was suspected. Two years later he developed progressive gait unsteadiness and frequent falls. On examination focal findings included reduction of upgaze, dysphasic language and slowness of gait with disequilibrium. A trial of carbidiopa/levodopa was unsuccessful. The diagnosis of probable PSP was suspected. On subsequent years he developed bulbar symptoms with dysartria, dysphagia and a behavior disorder with irritability, insomnia and occasional visual hallucinations (VH). He died from aspiration pneumonia 3 years and 9 months after the first visit.

### Histopathological Findings

The presence of α-synuclein aggregates was only found in 2 patients from our cohort comprising a total of 25 cases available at our Navarra Biomed Brain Bank. In both cases the immunohistochemical detection of phosphorilated tau demonstrated numerous positive neuronal and glial fibrillary inclusions including tufted astrocytes and coiled bodies, as well as neuropil threads fulfilling the neuropathological criteria of PSP (Hauw et al., 1994; Litvan et al., 1996) such as dystrophic axons and neurites. The greatest concentration of tau pathology was found in the SNc and in the LC. The subthalamic nucleus, the dentate nucleus of the cerebellum and pontine nuclei displayed a moderate density of tau pathology. Mild density of tau deposits were found in the hippocampus/entorhinal cortex (without a preferential layer distribution), putamen, pallidum and amygdaloid complex. Neocortical sections showed rare tau-positive glial and neuronal inclusions.

Regarding α-synuclein deposits, protein aggregates in the form of LBs and LN were consistently found throughout the brain stem, with particular high incidence at the level of the LC in both cases, as shown in Table 1. Mild density of LBs and LN were found in the motor dorsal vagal nucleus, raphe nucleus and SNc. Case 1 showed moderate density of α-synuclein deposits in the entorhinal cortex and amygdale while case 2 contained scattered LBs and LN in those areas. Both cases displayed mild α-synuclein deposits in the hippocampus dentate gyrus, cingulate cortex and nucleus basalis of Meynert. No LBs nor LN were found in the subthalamic nucleus, striatum, pallidum, thalamus or neocortical regions.

**Table 1:** **Density and distribution of Lewy bodies**.

Age/Sex	Brain weight		Cc	Hdg	Ec	A	nbM	SNc	LC	Rn	DMVn	PD Braak stage
84F	1050	LB	+	0	++	++	0	+	+++	+	+	4
		LN	0	+	0	+	+	+	++	0	+
73M	1200	LB	+	0	+	+	+	+	+++	+	N.A.	4
		LN	0	+	0	0	+	0	+	+	N.A.

*Abbreviations: A, amygdala; Cc, cingulate cortex; DMVn, dorsal motor vagal nucleus; Ec, entorhinal cortex; Hdg, hippocampus dentate gyrus; LC, locus ceruleus; N.A., not available; nbM, basal nucleus of Meynert; PD, Parkinson’s disease; Rn, raphe nucleus; SNc, substantia nigra pars compacta*.

**Figure 1:**
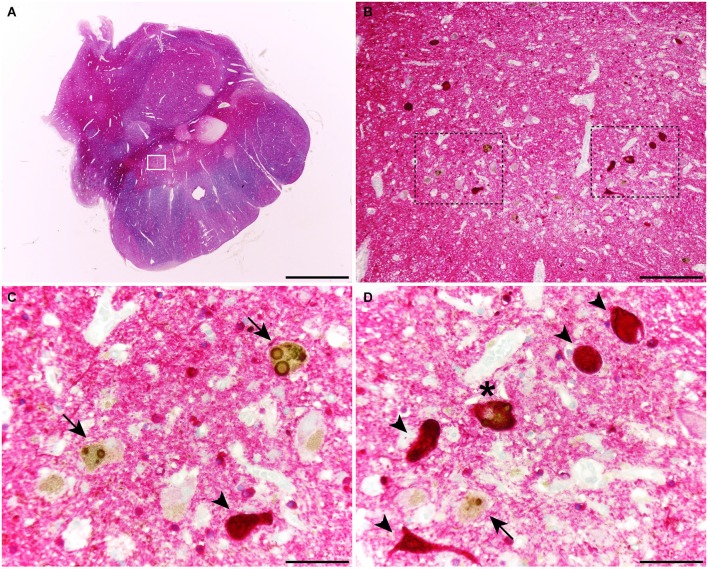
**Coronal section through the ventral mesencephalon showing the obtained dual stain for α-synuclein (TH; brown) and tau (red)**. At the level of the substantia nigra pars compacta (SNc), up to three different types of melanin-containing neurons were observed, comprising (i) brown-stained neurons containing Lewy bodies (LBs; arrows), (ii) red-stained neurons with tau immunoreactivity (arrowheads); and (iii) neurons showing both LBs and tau deposits (asterisks). Scale bar is 4,000 μm in **(A)**; 200 μm in **(B)** and 50 μm in the high-magnification insets shown in panels **(C)** and **(D)**.

**Figure 2:**
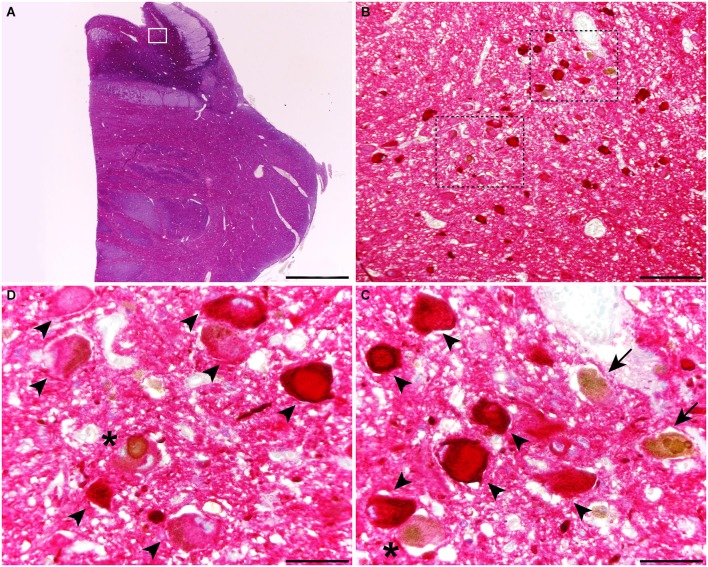
**Coronal section through the dorsal pons at the level of the Locus ceruleus (LC), stained for α-synuclein and tau**. At the level of the LC, up to three different types of melanin-containing neurons were observed, comprising (i) brown-stained neurons containing Lewy bodies (LBs; arrows), (ii) red-stained neurons with tau immunoreactivity (arrowheads); and (iii) neurons showing both LBs and tau deposits (asterisks). Scale bar is 4,000 μm in **(A)**; 200 μm in **(B)** and 50 μm in the high-magnification insets shown in panels **(C)** and **(D)**.

**Figure 3:**
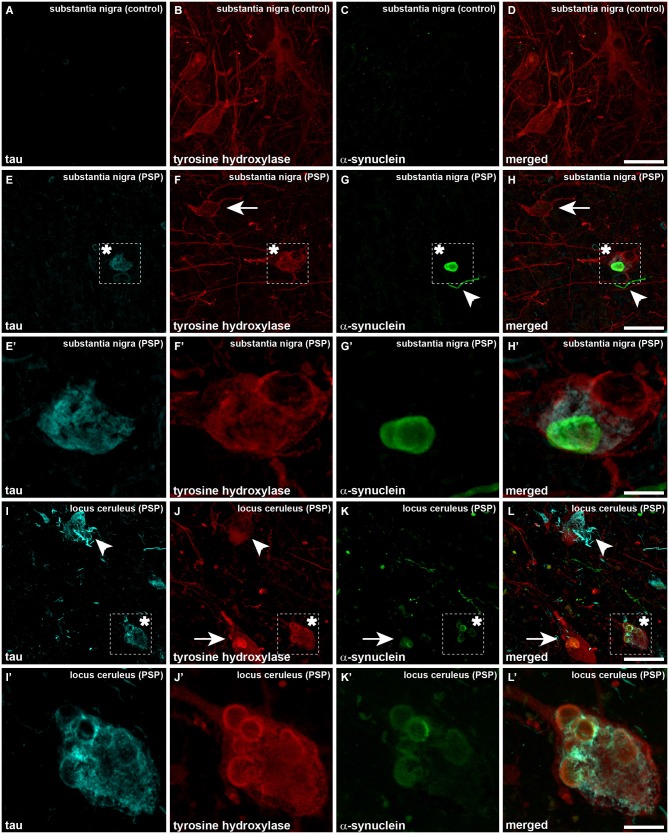
**Triple immunofluorescent detection of TH, α-synuclein and tau, as observed in a control case (A–D) as well as in the SNc and LC from a case diagnosed with PSP (E–L’)**. Neurons in the SNc from a control case are only immunoreactive for TH (red channel), without any noticeable immunoreactivity for tau and α-synuclein (**A** and **C**, respectively). When considering PSP cases at the level of the SNc and besides some neurons single-expressing TH (arrows), neurons containing both LBs and tau aggregates were clearly visible (asterisk). The presence of dystrophic neurites positive for α-synuclein is also often observed (arrowheads). The same holds true at the level of the LC. Panels **(I–L)** are low-magnification photomicrographs showing (i) TH+ neurons with tau aggregates (arrowheads), (ii) TH+ neurons with LBs (arrows); and (iii) TH+ neurons containing both LBs and tau immunoreactivity (asterisk). It is worth noting the presence of dystrophic neurites immunoreactive for either tau or α-synuclein. Panels **(I’,J’)** are high-magnification insets taken from **(I–L)** to better appreciate one TH+ neuron showing tau immunoreactivity and multiple LBs. Scale bar is 50 μm for low-magnification photomicrographs and 12.5 μm for insets.

When considering catecholaminergic nuclei such as the SNc and the LC, the dual colorimetric detection of tau and α-synuclein showed different types of neuromelanin-containing neurons, comprising (i) neurons with tau deposits; (ii) neurons with LBs (quite often with more than one LB); and (iii) neurons with both types of aggregates, i.e., tau and LBs (Figures 1, 2). These three types of neurons were also observed in the conducted triple immunofluorescent stains, which also fully confirmed the monoaminergic nature of these neurons by showing that all these types of neurons were also positive for TH, both at the level of the SNc and the LC (Figure 3). Furthermore, it is also worth noting that a minimal number of TH+ neurons completely lacked any type of protein aggregates (Figure 3).

## Discussion

Here, LBs were found in 8% of PSP brains, a percentage similar to what might be expected in series of age-matched normal controls (Tsuboi et al., 2001). The frequency of LBs in our PSP population was lower than in previous reports (Table 2). Besides case reports describing the incidental presence of LBs in PSP (Mori et al., 1986; Fearnley et al., 1991; Judkins et al., 2002; Abhinav et al., 2011) the percentages of LBs in necropsy series of PSP-diagnosed patients ranged between 10.7% to 31.5% (Tsuboi et al., 2001; Mori et al., 2002; Uchikado et al., 2006; Keith-Rokosh and Ang, 2008).

**Table 2:** **Studies analyzing the presence of Lewy bodies in progressive supranuclear palsy**.

Reference	N (%)	α-synuclein immunohistochemistry	Doble immunolabeling techniques	Neuronal colocalization of tau and α-synuclein
Abhinav et al. (2011)	1	Yes	No	-
Keith-Rokosh and Ang (2008)	4 (12,5)	Yes	No	-
Uchikado et al. (2006)	29 (10,7)	Yes	Yes	Yes
Judkins et al. (2002)	1	Yes	No	-
Mori et al. (2002)	5 (31,5)	Yes	Yes	Yes
Tsuboi et al. (2001)	13 (12)	Yes	Yes	Yes
Gearing et al. (1994)	2	No	No	-
Fearnley et al. (1991)	1	No	No	-
Mori et al. (1986)	1	No	No	-

In the two PSP cases of the present study, LBs were widely distributed throughout the brainstem and cerebrum in a pattern that looks like what can be expected in PD. These similarities on the distribution of LBs comparing PD and PSP have also been already reported, although some controversies remain, since it has been described that LBs were only found in the amygdala of PSP patients (similarly to AD brains) and not in neither the SNc nor the LC nucleus (Tsuboi et al., 2001; Uchikado et al., 2006). The two PSP patients described in this paper presented clinical manifestations of the classical phenotype of PSP, originally described by Richardson which accounts for the most common clinical form of PSP. The Richardson’s syndrome (RS) is characterized by an insidious onset and relentlessly progressive postural instability and falls, gait disturbance, supranuclear vertical gaze abnormalities, pseudobulbar palsy, rigidity in extension and a dysexecutive syndrome (Williams et al., 2005). The clinical significance of LBs cannot therefore be fully ascertained, bearing in mind that LBs might represent an incidental finding without any clinical significance (Tsuboi et al., 2001). It is also worth noting that PSP cases with LBs previously reported in the literature had often received clinical diagnosis other than PSP (Keith-Rokosh and Ang, 2008).

The second patient developed VH, a common finding in patients with underlying LB pathology considered to be due to neuronal dysfunction specific to α-synuclein accumulation (Popescu et al., 2004). The brain of this patient contained isolated LBs in the amygdala and in the cingulated cortex. By contrast, the brain from the first patient showed a higher density of LBs in the amygdala but she did not developed VH. Although infrequently observed, VH may also be present in PSP brains without LBs (Bertram and Williams, 2012). The patient described by Compta et al. (2009) with clinical features of PD and VH and widespread phosphorylated tau deposits consistent with PSP, included severe tau pathology in the hippocampus and amygdala in the absence of LBs, therefore leading to the suggestion that the location of pathological lesions in the brain is the most important factor determining VH (Compta et al., 2009). Although it is tempting to speculate that the clinical heterogeneity seen in parkinsonian disorders may reflect the occurrence of combined pathology, it is very likely that the distribution of pathological deposits also is a key underlying factor in determining clinical differences.

The main limitation of the present study is represented by the fact that LBs were only found in two PSP patients, therefore minimizing potential clinico-pathological correlates. Nevertheless, it should be stressed that our data unequivocally showed the presence of tau and α-synuclein co-expression within single TH-positive neurons in both the SNc and the LC nuclei. Earlier data from the literature have reported tau and α-synuclein co-localization in very few neurons of the nucleus basalis of Meynert and in the LC, whereas this co-expression was never observed in the SNc (Uchikado et al., 2006). Furthermore, a kind of “globular” α-synuclein aggregates in just a few neurons with phosphorylated tau-positive cytoplasm at the level of the pontine tegmentum has also been reported elsewhere (Tsuboi et al., 2001).

The colocalization of tau and α-synuclein in the same neuron suggests an association between tau and α-synuclein aggregation that could be mediated by tau inclusions that promote the fibrillation of α-synuclein to form LBs in regions with abundant tau lesions (Popescu et al., 2004). Evidence of an interaction between tau and α-synuclein at a biochemical level has also been found. Brain α-synuclein accumulation determined by western blotting was above normal levels in the substantia nigra of some patients with PSP (Tong et al., 2010). Experimental studies have demonstrated that coincubation of tau and α-synuclein synergistically promotes fibrillization of both proteins in LBs (Giasson et al., 2003) and interactions between α-synuclein and tau at the cellular level cause disruption of cytoskeletal organization, axonal transport defects and aberrant synaptic organization that contribute to neuronal dysfunction and death (Roy and Jackson, 2014). Moreover, it is worth noting that protein aggregation develops naturally with age, although the question of whether this aggregation is cause or consequence of normal -or abnormal- aging remains to be fully elucidated (Cuanalo-Contreras et al., 2013).

Double-label studies demonstrated that in Pick disease -a 3R tauopathy-, LBs usually colocalize with tau-positive Pick bodies (Popescu et al., 2004). Regarding AD cases, in which tau is composed of a mixture of 3R and 4R tau isoforms, LBs typically colocalize with tau-positive neurofibrillary tangles especially in neuronal populations vulnerable to both neurofibrillary tangles and LBs, such as those in the LC and basal nucleus of Meynert (Ishizawa et al., 2003). Moreover, a potential α-synuclein and tau co-aggregation in neurons and neuritis from the olfactory bulb in AD patients has been more recently reported (Fujishiro et al., 2008).

We conclude that the presence of LBs in PSP patients may be viewed as a secondary phenomenon probably reflecting a synergistic effect between α-synuclein and tau. This phenomenon is more evident in populations of catecholaminergic neurons such as dopaminergic neurons in the SNc and noradrenergic neurons at the level of the LC. Finally, bearing in mind the low incidence of α-synuclein and tau co-localization in our series of PSP patients, we strongly believe that a direct clinical correlate to be induced by the co-aggregation of these two misfolded proteins is very unlikely.

## Conflict of Interest Statement

The authors declare that the research was conducted in the absence of any commercial or financial relationships that could be construed as a potential conflict of interest.
